# Strategic Framework for Strengthening Health Laboratory Services in the COVID-19 Pandemic in Iran

**DOI:** 10.30699/IJP.2024.2013702.3192

**Published:** 2024-02-15

**Authors:** Nooshafarin Safadel, Parisa Dahim, Siamak Mirab Samiee, Katayoon Khodaverdian, Maryam Mir Mohammad Ali Roodaki, Soghra Anjarani, Shahla Farsi, Mahdi Rafiee, Marjan R. Farzami

**Affiliations:** 1 *Reference Health Laboratory of Iran, Ministry of Health and Medical Education, Tehran, Iran*; 2 *Cardiogenetic Research Center, Rajaie Cardiovascular Medical and Research Institute, Tehran, Iran*

**Keywords:** Clinical laboratory services, COVID-19 pandemic, COVID-19 nucleic acid testing, SARS-CoV-2 infection, Strategic planning, Laboratory diagnoses

## Abstract

**Background & Objective:**

Providing equitable access to good quality, timely, and affordable laboratory testing has always been a top priority for the Ministry of Health and Medical Education (MoH-ME) and the Reference Health Laboratory (RHL). Considering the significant role of medical laboratories in disease surveillance, RHL developed a strategic plan to manage laboratory services during the COVID-19 pandemic based on the “Strategic Framework for strengthening health laboratory services, 2016-2020” proposed by the World Health Organization (WHO). This article describes the steps taken to establish the strategic framework in Iran.

**Methods:**

Firstly, a National Laboratory Committee was formed in MoH-ME and a situation analysis was conducted to explore the strengths, weaknesses, opportunities, and threats in different components of our laboratory system. Gaps and resources needed to address those gaps were determined; then, RHL outlined operational processes and mechanisms for monitoring the activities.

**Results:**

The WHO strategic roadmap and its six strategic goals concerning leadership, quality, human resources, safety and security, laboratory networking, and rational use of laboratory testing, helped us to promote national laboratory services in accordance with health system requirements in the COVID-19 pandemic.

**Conclusion:**

The establishment of a national molecular laboratory network with more than 500 laboratories from different sectors may result in timely access to countrywide laboratory services and would be beneficial for future COVID-19 and/or other viral outbreaks. Continual evaluation of the COVID-19 laboratories' performance, production of PCR test kits by the local manufacturers, and development of a platform for virtual training would be other accomplishments that Iran achieved in coping with the recent pandemic.

## Introduction

The SARS-CoV-2 was first detected in December 2019 in Wuhan, China. The coronavirus disease 2019 (COVID-19) spread rapidly worldwide and was identified as a significant public health concern by WHO. The rapid spread of COVID-19 infection was associated with fundamental changes in the healthcare systems in different countries. The prevalence of COVID-19 in 215 countries worldwide prompted governments to develop national strategies tailored to their social, political, economic, cultural, and even religious conditions while adhering to international strategies (1).

On February 19, 2020, Iran health authorities confirmed two patients infected with SARS-CoV-2 in Qom city. More cases were reported in Tehran a few days later and the newly emerged coronavirus rapidly spread throughout the country (2). To manage the pandemic as efficiently as possible, the Ministry of Health took several critical measures. Considering the significant role of laboratory services in the detection of infected individuals, providing nationwide access to laboratory services was set as a top priority for the healthcare systems (3).

The Reference Health Laboratory (RHL) is a directorate-general in MoH-ME authorized for policy-making and strategic planning for the health laboratory system at the national level. One of the RHL's responsibilities is to ensure the availability of efficient laboratory services in disasters, crises, and emergencies. In this regard, important measures have been taken by RHL over the past few decades including management of the laboratory system preparedness and response in seasonal influenza outbreaks by predicting and providing necessary infrastructures and defining work processes and responsibilities. In the beginning, these arrangements helped us to respond to the COVID-19 pandemic; however, considering the rapid dissemination of the SARS-CoV-2, a systematic approach was needed to coordinate all the stakeholders and integrate laboratory services nationwide to cope with the situation during the pandemic. To do so, RHL followed the WHO strategic framework and its six interrelated strategic goals as a roadmap (4).

The purpose of this article was to describe how RHL developed an effective strategy based on the WHO framework and established action plans to ensure that health laboratory services are comprehensive, well-coordinated, integrated, and sustainable.

## Material and Methods

For integrated management of the laboratory services during the COVID-19 pandemic, a National Laboratory Committee was established in MoH-ME in February 2020 shortly after diagnosis of the COVID-19 cases in our country. RHL along with “The Pasteur Institute of Iran”, “The Public Health Deputy of MoH-ME”, “Food, Drug and Medical Equipment Reference Laboratories”, “The National Medical Device Directorate”, and “The Board of Trustees for Patients Treatments with Currency Saving “from the members of that committee. Roles and responsibilities of all members were defined, and tasks were assigned.

RHL's responsibility was to streamline the structures and functions of the national laboratory services to meet the health system's needs during the COVID-19 pandemic. To this purpose, a rapid situation analysis was conducted, and information was gathered from all around the country to explore the strengths, weaknesses, opportunities, and threats (SWOT) in different components of our laboratory system. Information concerning the number and distribution of molecular diagnostic laboratories in each province, their facilities, list of equipment, and technical experts were collected and reviewed; in this way, we estimated actual and potential molecular testing capacity in the country. Additionally, the availability of the resources and supplies required to maintain and expand laboratory services were meticulously considered as well as the threats mainly caused by the strict sanctions imposed on our country.

To manage the laboratory services during the pandemic, RHL decided to pursue the “Strategic Framework for strengthening laboratory services” introduced by WHO and establish its six strategic goals, which are as follows:

1. Strengthen leadership and governance of the national laboratory systems.
2. Strengthen the organization and management of the national laboratory systems towards quality.
3. Establish sustainable, sufficient, and competent human resources for the laboratory service delivery.
4. Ensure safe and secure laboratory environments.
5. Promote effective laboratory referral networking and enhance coordination.
6. Promote rational and evidence-based use of the laboratory service.

The realistic objectives were devised based on the SWOT analysis results. To achieve the objectives, RHL set up detailed action plans and defined operational processes and procedures for monitoring and evaluation of the activities. Allocation and/or distribution of adequate resources to implement the action plans were also taken into consideration.

## Results

The establishment of the WHO strategic framework led to remarkable achievements, most importantly expanding the number of laboratories from less than 15 molecular laboratories at the beginning of the pandemic to about 500 laboratories. The establishment of a molecular laboratory referral network enabled unrestricted access to laboratory services throughout the country. Regulatory processes were defined and ongoing assessment plans such as on-site inspections and specialized External Quality Assessment Scheme (EQAS) were established for licensing and re-licensing of the COVID-19 laboratories.

Testing capacity increased from less than 150 molecular diagnostic tests per day up to 70,000 tests per day by the end of the pandemic. The turnaround time of the COVID-19 molecular tests reached less than 24 hours on average, while it was more than 72 hours at the beginning of the pandemic.

Production of SARS-CoV-2 RT-PCR and Antigen-Rapid Diagnostic Test (Ag-RDT) kits by the local manufacturers reduced the COVID-19 testing costs and increased the affordability of the laboratory services resulting in increasing COVID-19 laboratory testing capacity to more than 200,000 tests per day.

National protocols and technical guidelines on safe and quality practice were developed and virtual training programs and webinars were implemented to improve the knowledge and skills of the laboratory staff. 

Production of personal protective equipment (PPE), triple package containers (for the safe transportation of infectious specimens), and safety cabins (for safe respiratory sampling) by the local manufacturers were promoted during the COVID-19 pandemic.

## Discussion

The COVID-19 pandemic was associated with fundamental changes in the healthcare system in different countries (1). Countries had to assess their risks and implement necessary measures at an appropriate scale to enhance their surveillance systems for the early detection, isolation, and laboratory confirmation of the suspected COVID-19 cases, to manage the spread of the pandemic and increase the level of preparedness to identify, manage, and care for the new cases of COVID-19 (5).

All countries realized that there were different public health scenarios with no “one-size-fits-all” approach for managing cases and outbreaks. Thus, each country considered different strategies and took different measures in dealing with the COVID-19 pandemic (6), such as building new hospitals (China) (7), designing and using patient tracking applications (Singapore) (8), designing and operating a COVID-19 screening system (Iran) (9), and implementing quarantine operations along with increasing capacity of the hospitals to minimize the challenges of the treatment system (Italy) (10). Some other strategies and measures adopted by other countries are as follows:

Containment measures in Turkey were composed of four essential strategies i.e., “testing”, “contact tracing”, “treatment” and “quarantine/ isolation”. Turkey also increased its daily testing capacity rapidly, reaching up to 40,097 per million in June 2020, one of the best ones in the region. Therefore, they were able to trace close contacts, isolate/ quarantine, and/or treat them; thus, interrupting transmission chains effectively (11).

In India, Indian Council of Medical Research (ICMR)-approved public and private laboratories helped to increase the public sector COVID-19 testing capacity to nearly 100,000-120,000 tests per day through the deployment of additional workforce, procurement of corresponding IVDs, and establishment of a sample referral system (12).

In Canada, the Ministry of Health established a COVID-19 Testing and Screening Expert Advisory Panel in November 2020. The Panel provided evidence-informed advice to the federal government on approaches to COVID-19 testing and screening. Given the diversity in geography, demographics, science, experience, and technologies available as well as domestic and international data, the panel suggested focusing on four priority areas including 1) optimizing the diagnostic capacity of laboratory-based PCR testing, 2) deploying rapid screening tests, 3) addressing equity considerations for testing and screening programs, and 4) improving communication strategies (13).

Germany responded to the COVID-19 pandemic by implementing a national program at the federal level by the Robert Koch Institute (RKI). The low rate of fatalities versus the high rate of infected patients indicates the successful policies adopted such as providing a large number of screening tests i.e. 500,000 tests per week, rapidly implementing social prevention methods, and providing a high bed-to-patient ratio (56,000 beds for infected patients with coronary artery disease) (1).

To manage the pandemic as efficiently as possible in Iran, MoH-ME took some critical measures namely determined referral hospitals for admission of COVID-19 patients, supplied medical equipment, and in vitro diagnostics (locally manufactured or imported) to meet the priority needs of the population. MoH-ME also developed and periodically updated national guidelines on diagnosis and treatment of the COVID-19 pandemic. 

RHL as one of the WHO collaborating centers, responsible for managing the nationwide laboratory services, was convinced that the implementation of WHO's “Strategic Framework for Strengthening Health Laboratory Services 2016-2020” would be a crucial platform for improving the health laboratory systems in all countries. RHL considered this framework as a road map to provide reliable and sustainable laboratory services during the COVID-19 pandemic. In this respect, RHL coordinated activities to implement the six strategic goals. The measures taken to attain each goal are as follows:

**Strengthen Leadership and Governance of the National Laboratory Systems**

To enforce the establishment of a COVID-19 molecular laboratory network:

Having reviewed the results of the SWOT analysis, the National COVID-19 Laboratory Committee concluded that laboratory services during the COVID-19 pandemic should be delivered through a laboratory network to make it possible to improve access to efficient diagnostic testing despite the limited resources. To this purpose, the capabilities of all molecular diagnostic laboratories in different sectors i.e. government and public health laboratories, private laboratories, academic laboratories, and research molecular laboratories were integrated, and the COVID-19 molecular laboratory network was established (see 5.1)

**Laboratory Licensing and License Renewal**

RHL defined certain regulatory processes to approve the molecular laboratories for joining the COVID-19 laboratory network. The purpose was to ensure that all laboratory members met the quality and safety standards.

Offices of laboratory affairs in all medical universities were assigned to perform on-site evaluations and send the assessment reports back to RHL. Qualified assessors inspected the candidate laboratories. Laboratories that meet the standards through on-site visits would register for the next step to participate in COVID-19 EQAS provided by the Pasteur Institute COVID-19 National Laboratory. Laboratories that succeeded in this program were considered to be technically qualified. Subsequently, they were required to sign a letter of commitment to declare that they were committed to the statutory rules and regulations. After taking all these steps, RHL issued a working license that allowed the laboratory to perform SARS-CoV-2 molecular testing as a member of the National COVID-19 Laboratory Network. An ongoing assessment plan was also in place. The office of laboratory affairs in medical universities was held responsible for the periodic assessment of these laboratories as part of their annual operational plan. The assessment reports were submitted to the “MoH-ME operational plan website”, which is accessible to RHL for supervision. It should be mentioned that the licenses were valid only for six months and would be renewed only if laboratories obtained satisfactory scores in ongoing COVID-19 EQAS. Otherwise, the license would be suspended or even canceled.

The Roaster of the approved laboratories was posted on the MoH-ME website and updated weekly.

**Management of Complaints**

Any complaint submitted against molecular diagnostic laboratories by their clients or legal authorities was either investigated and addressed by RHL or referred to the relevant authorities. If violation of the regulations was proven, the laboratory had to pay a certain amount of fine. In serious cases, it might lead to the license revocation.

**Supply Chain Management**

At first, only imported COVID-19 test kits were available in the market. Over time, local manufacturers started producing SARS-CoV-2 RT-PCR and Ag-RD test kits. “The National Medical Device Directorate” and “Board of Trustees for Patients Treatments with Currency Saving” had a key role in the nationwide supply of approved equipment and IVDs, either locally manufactured or imported. RHL was responsible for the need assessment, centralized procurement, and proper distribution of the IVDs for the laboratories in the public sector. It is worth mentioning that Pasteur Institute COVID-19 National Laboratory as well as “Food, Drug and Medical Equipment Control Reference Laboratories” were responsible for the premarket evaluation of these equipment.

**Tariffs for Laboratory Testing**

The COVID-19 molecular testing was first free of charge in some public laboratories only for symptomatic patients and suspicious cases according to the MoH-ME protocols. At the beginning of the pandemic, molecular tests were expensive and barely available in private laboratories since there were only imported SARS-CoV-2 RT-PCR test kits in the market; as domestic COVID-19 test products increased and saturated the market, the PCR test tariff was cut almost by half.

Currently, all private laboratories must charge people according to the approved tariffs; otherwise, the laboratory will be penalized and may even lose its license; additionally, designated public laboratories perform the molecular tests free of charge if it is indicated by the national COVID-19 surveillance protocols.

**Laboratory Information Management**

Collecting and analyzing laboratory data will yield valuable information that could demonstrate how countries are coping with the virus and whether they adopt appropriate strategies to manage the pandemic. Laboratory information could also be used for further evidence-based decision-making and well-informed management of the health services in the COVID-19 pandemic. Thus, all COVID-19 testing laboratories in different public and private sectors are required to follow national reporting regulations. Test results either positive or negative should be immediately submitted as directed by the health authorities.

From the beginning of the pandemic, the COVID-19 National Laboratory Committee emphasized establishment of the integrated laboratory information systems for the timely and accurate recording of COVID-19 laboratory test results. In this regard, MoH-ME developed several information system software for the registration of laboratory results obtained from hospitalized patients as well as outpatients referring to the public and private sectors (3).

**Conducting the National Laboratory System Toward Quality**

**Providing national guidelines**

National protocols and technical guidelines closely aligned with and/or adapted from WHO guidelines (14,15,16,17) were developed mandatory to be followed by all the COVID-19 molecular diagnostic laboratories. Practical guidance was also documented and published for all stakeholders using COVID-19 Ag-RDT and Antigen ELISA tests.

**Periodic On-site Inspections**

Strict quality-related regulations were set for the laboratories' licensing and license renewal. All licensed COVID-19 laboratories are inspected at least twice a year. To facilitate and harmonize the assessment process, a specialized checklist was drawn up based on the WHO “Molecular Laboratory Checklist” being used as assessment criteria (18).

**Implementing SARS-CoV-2 EQAS**

All COVID-19 molecular laboratories are required to participate in SARS-CoV-2 EQAS provided by the Pasteur Institute COVID-19 National Laboratory every 6 months. Panels of five unknown specimens are sent to the designated laboratories and they are required to perform the molecular tests and send the results back to the Pasteur Institute National Laboratory. The results are shared with RHL. Only laboratories that obtain passing scores are considered competent to start and/ or continue SARS-CoV-2 testing. It is noteworthy that the COVID-19 national laboratory at the Pasteur Institute of Iran has been responsible for providing technical support and troubleshooting for the COVID-19 molecular laboratory network all around the country.

**Establishing Sustainable, sufficient, and competent human resources**

**Improving the Knowledge and Skills of Laboratory Personnel**

It is crucial to ensure that laboratory personnel are well-trained in biosafety and biosecurity requirements, and competent in technical skills to perform the work in the pandemic (1). To this purpose, RHL prepared training packages on quality and safety and launched virtual training programs and webinars to make it possible for all staff involved in the COVID-19 testing services, even in remote provinces, to have access to the electronic training modules.

**Increasing the Number of Laboratory personnel**

Taking into account the growing need for widespread COVID-19 molecular testing and increasing laboratory workload, personnel with a broader range of laboratory-related education were recruited and trained to be able to cover the excessive workload.

**Promoting Staff Motivation**

Various strategies were considered for the personnel retention and increasing their motivation such as competitive pay and benefits for the staff working in COVID-19 molecular testing departments; however, it has not been implemented equally in different provinces yet and it remains to be one of our challenges.

**Ensuring Safe and Secure Laboratory Environments**

**Biosafety and Biosecurity Guidelines**

RHL had already developed and enforced laboratory biosafety and biosecurity requirements as a part of our national laboratory standards. Moreover, a big section of our comprehensive assessment checklist is dedicated to safety requirements, thus, laboratory assessors regularly evaluate laboratories against those requirements. During the COVID-19 pandemic, specialized guidelines and virtual training packages were developed on different safety-related topics such as safe nasopharyngeal and oropharyngeal sampling and transportation of the specimens, donning, and doffing of PPEs, spill management, and waste management for the COVID-19 testing laboratories. Meanwhile, RHL translated the “WHO guidance on regulations for the transport of infectious substances, 2021-2022” and emphasized that all procedures must be performed based on the risk assessment and only by qualified personnel in accordance with the relevant protocols. 

**Locally Manufactured Safety Equipment**

Local manufacturers were guided and supported to scale up production of personal protective equipment (PPE), triple package containers (for the safe transportation of infectious specimens), and safety cabins (for safe respiratory sampling) during the COVID-19 pandemic.

**Promoting Effective Laboratory Referral Networks and Enhancing Coordination**

**COVID-19 Molecular Laboratory Network**

At the beginning of the COVID-19 pandemic, there were only two laboratories, which set up the molecular diagnostic testing for the newly emerged coronavirus and all suspicious cases were referred to them. With the growing number of COVID-19 cases, the COVID-19 testing laboratory services had to be expanded throughout the country. The COVID-19 National Laboratory Committee recommended that a laboratory network be organized to provide optimal access to the COVID-19 molecular diagnostic services.

Several laboratory diagnostic networks and specimen referral systems were already developed to provide countrywide laboratory services for under-surveillance diseases such as HIV, influenza, TB, and so on. Moreover, RHL implements “Specimen Referral Maneuvers” on an annual basis to evaluate the operational capabilities of the public health laboratories for the safe and timely transportation of infectious specimens.

This was a valuable experience useful for the establishment of the COVID-19 molecular laboratory network. In doing so, firstly, RHL demanded fifteen public health laboratories in fifteen provinces (already performed influenza and HIV molecular tests) to set up SARS-CoV-2 molecular diagnostic methods. Academic and research molecular laboratories were also encouraged to launch COVID-19 molecular testing and start providing public services. As COVID-19 affected larger areas of the country, RHL planned to use the capacity of laboratories in private sectors to expand the laboratory network. Private laboratories, that managed to fulfill the professional standards and regulatory requirements, were granted a license to join the COVID-19 molecular laboratory network. Establishment of the service maps and COVID-19 specimen referral systems from “Sampling Facilities” to designated laboratories to ensure access to laboratory services in remote areas of the country.

 “Iran COVID-19 Emergency Response Projects” (ICERP-1) (19), joint projects carried out by Iran MoH-ME and WHO, also strengthened the COVID-19 laboratory network. Under RHL supervision, selected molecular testing devices were purchased and distributed to the 43 central public health laboratories in 31 provinces to join the COVID-19 molecular laboratory network. Next Generation Sequencing (NGS) technology deployed in national and referral laboratories in the COVID-19 laboratory network was used to identify SARS-CoV-2 variants and provide epidemiological information for disease surveillance.

The establishment of a nationwide network of COVID-19 molecular diagnostic laboratories with more than 550 laboratories from the public and private sectors was a valuable achievement that enabled our health system to provide timely access to quality laboratory services during the COVID-19 pandemic (3).

**Using Antigen Rapid Diagnostic Testing (Ag-RDT) Methods to Increase Testing Capacity**

 Nucleic acid testing (NAT) using reverse transcription-polymerase chain reaction (RT-PCR) is the most sensitive and specific method and gold standard for diagnosing COVID-19 (20), however, RT-PCR assays are expensive, take several hours to generate results, and require complex laboratory equipment and trained technicians. As the pandemic continued, SARS-CoV-2 Ag-RDT methods got approval and became available with much lower prices, less complexity, and less turnaround time than the reference method. Despite its lower sensitivity, Ag-RDT offers an opportunity to increase the availability and speed of testing in appropriate scenarios (21). For instance, Ag-RDT was used in a family/ neighborhood-centered national project to find infected people who were asymptomatic or pre-symptomatic. Additionally, contact tracing using Ag-RDT has been performed to ensure that individuals who knowingly or unknowingly contacted COVID-19 patients could be diagnosed, isolated, and treated promptly. The establishment of a COVID-19 molecular laboratory network and specimen referral systems along with deploying rapid SARS-CoV-2 Ag tests, resulted in increasing COVID-19 laboratory testing capacity to more than 200,000 tests per day at present.

**Promoting Rational and Evidence-based Use of Laboratory Services**

Indications for COVID-19 test ordering should be clear especially if resources are limited. Most countries have used clinical and epidemiologic information to determine who should be tested; therefore, strategies for active case finding and contact tracing vary from one country to another. Testing strategies are especially important as countries initiate easing COVID-19 restrictions.

COVID-19 testing protocols advised by MoH-ME have periodically been changed since the beginning of the pandemic. RHL cooperated in developing MoH-ME guidelines on rational COVID-19 test ordering and utilizing the laboratory services in the pandemic for diagnosis and follow-up of symptomatic patients, suspicious individuals, and contact tracing as well as COVID-19 screening for travelers crossing the borders. The guidelines explain the indications for performing laboratory tests, interpretation of the results, and limitations of different COVID-19 test methods including molecular assays and Ag detection methods. However, feedback revealed that not all healthcare workers followed those guidelines, and test requests were submitted out of protocol.

## Conclusion

WHO's strategic framework and its six strategic goals for strengthening the health laboratory services inspired RHL to draw up a roadmap and manage the laboratory services during the COVID-19 pandemic more effectively.

The establishment of a molecular laboratory network in a short while was a great achievement for our health system that enabled nationwide access to laboratory services. It is noteworthy that the molecular network provides a remarkable capacity for the future surveillance of other epidemic-prone diseases such as CCHF, Dengue fever, Monkeypox, Chikungunya, and so on. Another opportunity caused by the COVID-19 pandemic was the development of an online learning platform, thus virtual training programs on priority topics have become accessible to the laboratory staff in deprived areas of the country. From then on RHL used this opportunity to rapidly share important information.

As for challenges, despite the importance of the laboratory information for further evidence-informed policymaking, not all laboratories managed to submit COVID-19 test results to designated software promptly. On the other hand, the multiplicity of laboratory information system software resulted in difficulty in data mining i.e. exploring and analyzing the collected data to turn raw data into useful information by finding trends and patterns.

Workforce sustainability becomes a serious challenge in providing laboratory services over time in a situation like the COVID-19 pandemic. RHL is planning to enhance staff motivation by establishing an employee reward system and financial incentives.

Irrational test ordering was another challenge during the pandemic. Underutilization and overutilization of laboratory services have largely been a threat to patient care and optimal use of resources. It has to be taken into consideration by the health authorities.

As improvement plans, we should focus on promoting inter-sectoral coordination and partnership, upgrading laboratory equipment and environmental conditions, especially in the public sector, and integrating laboratory information systems. Addressing those issues could improve our laboratory response to future unforeseen crises and emergencies.
